# Association between psychological distress of each points of the treatment of esophageal cancer and stress coping strategy

**DOI:** 10.1186/s40359-022-00914-5

**Published:** 2022-09-06

**Authors:** Yu Ohkura, Kanako Ichikura, Junichi Shindoh, Masaki Ueno, Harushi Udagawa, Eisuke Matsushima

**Affiliations:** 1 Section of Liaison Psychiatry and Palliative Medicine, Graduate School of Medical and Dental Sciences, Tokyo Medical and Dental University, Tokyo, Japan; 2 Department of Gastroenterological Surgery, Toranomon Hospital, 2-2-2 Toranomon, Minato-ku, Tokyo, 105-8470 Japan; 3 Okinaka Memorial Institute for Medical Research, Tokyo, Japan; 4 Department of Health Science, School of Allied Health Sciences, Kitasato University, Kanagawa, Japan; 5 Department of Gastroenterological Surgery, Digestive Tract Center, Toranomon Hospital Kajigaya, Kanagawa, Japan

**Keywords:** Psychological distress, Coping, Anxiety, Depression, Esophageal cancer

## Abstract

**Background:**

Patients with esophageal cancer often feel depressed and are fearful of metastasis and death. Esophagectomy is an invasive procedure with a high incidence of complications. The objective of this study was to examine the association between psychological distress on each points of the treatment of esophageal cancer and stress coping strategy.

**Methods:**

In total, 102 of 152 consecutive patients who attended the outpatient clinic at Toranomon Hospital between April 2017 and April 2019 met the eligibility criteria for inclusion in this study. Questionnaires designed to identify psychological distress and stress coping strategies were longitudinally administered at 5 time points from the time of the first outpatient consultation to 3 months after esophagectomy.

**Results:**

Although ‘fighting spirit’ (OR 0.836, 95% CI 0.762–0.918; *p* < 0.001) and ‘anxious preoccupation’ (OR 1.482, 95% CI 1.256–1.748; *p* < 0.001) were strongly related to psychological distress before treatment, as time of treatment passes, ‘helpless/hopeless’ (OR 1.337, 95% CI 1.099–1.626; *p* = 0.004) was strongly related to psychological distress after esophagectomy. There were no relationships between psychological distress and individual patient characteristics, with the exception of ‘history of surgery’ and ‘final staging’. The concordance index was 0.864 at time 1, 0.826 at time 2, 0.839 at time 3, 0.830 at time 4, and 0.840 at time 5.

**Conclusions:**

The relationship between psychological distress and the Coping strategies was stronger on each points of the treatment of esophageal cancer than that between psychological distress and individual patient characteristics. This study uses prospective basic clinical data and may provide the baseline information for risk stratification for psychological management and for future clinical studies in these patients.

**Supplementary Information:**

The online version contains supplementary material available at 10.1186/s40359-022-00914-5.

## Background

Esophageal cancer is the sixth leading cause of cancer-related mortality worldwide because of the high malignant potential and poor prognosis [1]. The postoperative 5-year survival rate in patients with American Joint Committee on Cancer stage I esophageal cancer is approximately 90%, and it decreases to 45% in patients with stage II disease, 20% in those with stage III disease, and 10% in those with stage IV disease [2–4]. In recent years, neoadjuvant therapy involving chemotherapy and/or radiotherapy is commonly used as an adjunct to surgical resection. Esophagectomy is the mainstay of curative treatment for esophageal cancer. However, it is a highly invasive procedure with a high incidence of complications and is one of the most complex interventions in gastrointestinal surgery. Recent advances in surgical techniques and perioperative intensive care have reduced the mortality and complications associated with esophagectomy [5], but it continues to be a challenging procedure with a reported mortality rate of 2.9–3.0% and a postoperative complication rate of 42.8–50.0% [6–8]. When looking at the incidences of postoperative morbidity rates, pneumonia, prolonged mechanical ventilation, need for transfusion, unplanned intubation, and systemic sepsis were relatively frequent (14.6%, 10.1%, 8.8%, 7.2%, and 7.0%, respectively) [8]. This study found that esophageal cancer surgery has a relatively high complication rate and is a high-risk surgery. Previous research on emotional outcomes after resection for esophageal cancer found that a substantial proportion of patients who were alive at 1 year felt depressed (64%) and expressed fear of metastasis and death (80%) [7]. We also described the relationship between psychological distress and health-related quality of life (HRQOL) in esophageal cancer [9]. Several reports have also been made on other types of cancer. It was reported that the prevalence of psychological distress varied according to the episode of the treatment of breast cancer [10]. In breast cancer patients, the coping response such as fighting spirits and helplessness/hopelessness were significant determinants of psychiatric morbidity [11]. In lung cancer patients, greater risk for depression was strongly associated with psychological factors, such as personality characteristics (neuroticism) and Coping strategies (low fighting spirit, helplessness/hopelessness, and anxious preoccupation) [12]. There is a possibility that these Coping strategies in each patients improves the psychological distress because there were significant associations between the Coping strategies and psychological distress [12–15]. However, it is still unclear whether the Coping strategies act on the psychological distress effectively in the various situations of the episode of the esophageal cancer treatment. These Coping strategies such as fighting spirits and helplessness/hopelessness in the past reports were important response to treatment for cancer, but these reports described only one point of the treatment for other cancers [11, 12, 15]. Past reports related to esophageal cancer described only one point of the treatment, too [16–18]. Before this study, we expected that there might be the appropriate Coping strategies on each points of the treatment of esophageal cancer. And also, in esophageal cancer patients, there were few studies that examined the risk factor and psychological reactions or conditions in each point of treatment in more detail from outpatient clinic to 3 months after surgery. Risk factors for psychological distress related to treatment for esophageal cancer were assessed statistically. We selected various variables which are the perioperative information including the operative findings accumulating regularly unintentionally at our hospital. We think that the patient’s psychological aspects differ on each point. We expected that the deleterious Coping strategy will influenced at almost all the situations, and the beneficial Coping strategies will influenced at the situation that important decisions are accomplished for patients. We also expected before this study that the patients with history of surgery might be hard to feel depressed than the patients without history of surgery. If we can describe the relation between perioperative psychological distress and the personal Coping strategies on various situations, it would be possible to develop appropriate supportive interventions and reduce the psychological stress, anxiety, and depressive feelings associated with esophageal cancer. Therefore, the aim of this study was to examine the association between psychological distress on each points of the treatment of esophageal cancer and stress coping strategy.

## Methods

### Study design and participants

A total of 152 consecutive patients with esophageal cancer who attended the outpatient clinic in the Department of Gastrointestinal Surgery at Toranomon Hospital between April 2017 and April 2019 were assessed for trial eligibility. Among these, 102 patients who met the eligibility criteria participated in this study. This sample size for this study is an convenient sample.

**Fig. 1:**
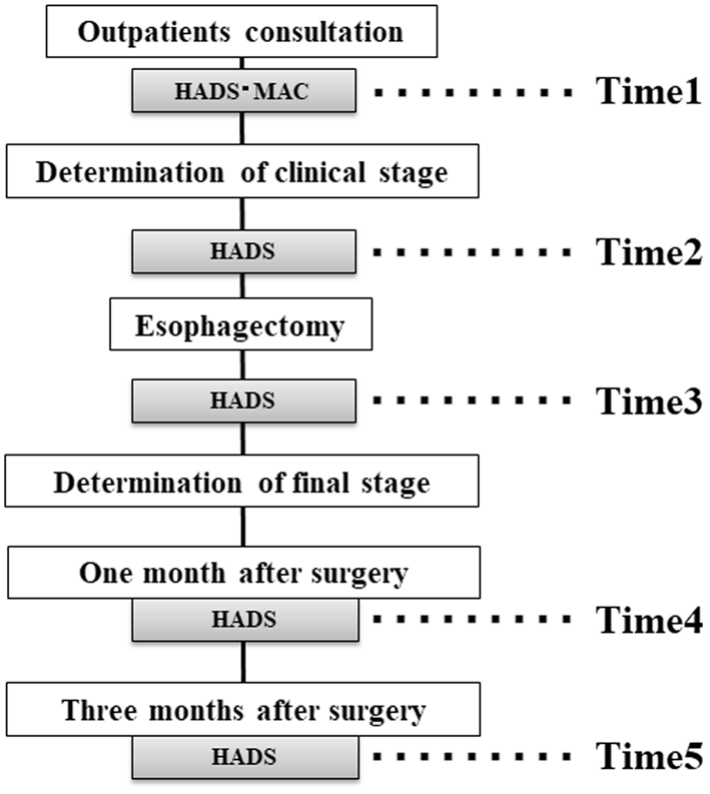
Flow of patients through the study. Time 1, before definitive diagnosis; time 2, after determination of clinical stage; time 3, postoperatively before final staging; time 4, determination of final stage at 1 month after esophagectomy; time 5, 3 months after esophagectomy

The inclusion criteria were as follows: a diagnosis of esophageal cancer, including Siewert type I/II esophagogastric junction tumors treated by subtotal esophagectomy; age 20–85 years; performance status 0–2; ability to provide informed consent; and awareness of the diagnosis of cancer. The following exclusion criteria were applied: informed consent not obtained; history of severe cognitive impairment or a psychiatric disorder; unsuitability for participation in the study because of psychological or physical stress in the opinion of the attending doctor or nurse; refusal to undergo surgery; and nonsurgical treatment. The flow of patients through the study is shown in Fig. 1.

Questionnaires were administered at 5 time points as follows: at the time of consultation in the outpatient clinic or on admission for examination to make a definitive diagnosis (time 1); during pre-treatment after determination of clinical stage (time 2); at 14 days postoperatively before final staging (time 3); at final staging (time 4); and 3 months after esophagectomy (time 5). The questionnaires were administered in the waiting room at our institution (Fig. 1). Disease stage was determined using the UICC TNM grading system, 7th edition [19]. All postoperative complications were graded using the Clavien-Dindo classification [20]; events more severe than grade ≥ III were recorded as complications.

The study protocol was approved by the institutional review boards of the Graduate School of Medical and Dental Sciences (approval number M2016-241) and Toranomon Hospital (approval number 1312) and registered with the UMIN Clinical Trials Registry (UMIN-CTR, R000033229). All procedures were conducted in accordance with the ethical standards of the Helsinki Declaration of 1975. Informed consent was obtained from all study participants at the time of the first outpatient appointment.

### Measures

#### Mental adjustment to cancer scale

We assessed the Coping strategies in this study using the Mental Adjustment to Cancer (MAC) scale. The MAC scale is a leading measure of coping and developed specifically for assessment of people with cancer. The questionnaire was developed to assess the extent to which patients adopt these specific Coping strategies in their adjustment to the diagnosis and treatment of cancer [11–15]. It was developed as a self-rating questionnaire that would be patient-friendly and could be administered easily at busy oncology clinics. The scale includes five subscales that measure five types of response: fighting spirit (“I firmly believe that I will get better”, 16 items); helpless/hopeless (‘I feel that life is hopeless”, 6 items); anxious preoccupation (“I suffer great anxiety about it”, 9 items); fatalism (“I’ve left it all to my doctors”, 8 items); and avoidance (“I don’t really believe I have cancer”, 1 item). Each item is scored on a 4-point Likert scale (1, “definitely does not apply to me”; 4, “definitely applies to me”). Scores for the subscales are calculated by summing the answers for the assigned items. The reliability and validity of the Japanese version MAC scales for Japanese cancer patients were confirmed by Akechi et al. [21]. Generally, the Coping strategies do not greatly change in the short time, therefore we didn’t measure the MAC scale at each time point [22, 23]. This questionnaire was administered once, at time 1, in this study.

#### Hospital anxiety and depression scale

This scale (HADS) is a 14-item self-rating tool designed to assess symptoms of anxiety and depression in medical patients, with an emphasis on reducing the impact of physical illness on the total score [24]. The HADS-total comprises two subscales that measure anxiety (HADS-A) and depression (HADS-D). Each subscale contains 7 items with scores ranging from 0 to 21 [24]. A total score of ≥ 11 on either subscale indicates a definitive case. We chose this cut-off value on the basis of the Japanese version of the Hospital Anxiety and Depression Scale (HADS), which has been validated for Japanese patients with cancer [25]; a cut-off HADS total score ≥ 11 has been recommended for identifying patients with potential adjustment disorder and major depression [25]. We defined a total score ≥ 11 as indicating psychological distress. The Japanese version of HADS was back-translated by Kitamura [26] and its reliability and validity was confirmed by Kugaya et al. [25]. The questionnaire was administered at all 5 time points in this study.

### Statistical analysis

Risk factors for psychological distress related to treatment for esophageal cancer were assessed by bivariate logistic regression analysis (Backward stepwise selection). We selected 31 variables which are the perioperative information including the operative findings accumulating regularly unintentionally at our hospital. Differences between the group with HADS scores ≤ 10 and the group with HADS scores ≥ 11 were tested for statistical significance using Fisher’s exact test, the unpaired Student’s *t*-test, the Mann–Whitney *U* test, and Pearson’s chi-squared test as appropriate. The variables with a *p*-value less than 0.05 in univariate analysis were entered into bivariate logistic analysis. The sets of bivariate logistic analysis were constructed in the development cohort using backward stepwise selection of predictors (a *p-*value < 0.05 was required for inclusion). Odds ratios (ORs) and their 95% confidence intervals (CIs) were calculated. A *p*-value less than 0.05 was considered statistically significant in bivariate logistic analysis. The discrimination ability of the model was assessed using receiver-operating characteristic (ROC) curve analysis, that is, the concordance (C)-index (area under the curve) values, at times 1–5. All analysis was performed using SPSS for Windows software (version 19.0 J; IBM Corp., Armonk, NY).

## Results

### Patient characteristics

Fifty of the 152 patents considered for participation in the study were excluded because they had incomplete data (n = 27), declined to participate (n = 11), did not undergo esophagectomy (n = 11), or were receiving treatment for a psychiatric disorder (n = 1), leaving 102 patients for inclusion in the study.

The characteristics of these 102 patients are shown in Table 1. Median age was 68.2 (range 44–86) years, 83.7% were male, and mean body mass index was 22.3; 23.5% had a history of cancer, 35.3% had a history of surgery, 86.3% had a history of alcohol consumption, and 85.3% had a history of smoking. Total HADS score was ≥ 11 points in 37 patients (36.3%) at time 1, 41 (40.2%) at time 2, 48 (47.1%) at time 3, 44 (43.1%) at time 4, and 35 (34.3%) at time 5. The MAC scale scores recorded at only time 1 are shown in Table 1.

**Table 1:** Patient demographics and clinical characteristics

Variables	Entire study population (N = 102)
Age, years, median (range)	68.2 (44–86)
Male sex, %	84.3
BMI, kg/m^2^, median	22.3 (14.1–41.9)
History of cancer	
Yes	24
No	78
History of surgery	
Yes	36
No	66
History of alcohol consumption	
Yes	88
No	14
History of smoking	
Yes	87
No	15
Brinkman index	612 (0–3040)
< 600	44
≥ 600	58
Thoracic approach	
Video-assisted thoracoscopic surgery	88
Open	9
None: transhiatal	5
Abdominal approach	
HALS	47
Open	24
Laparoscopic	31
Lymphadenectomy	
D0	2
D1	2
D2	27
D3	71
Curability	
R0	96
R1/2	6
Reconstruction	
Gastric tube	66
Ileocolic	26
Other	10
Thoracic duct	
Resected	62
Preserved	40
Reconstruction route	
Retrosternal	88
Posterior mediastinal	14
Preoperative treatment	
Yes	65
No	37
Operation time (min)	592 (213–774)
Blood loss (mL)	205 (25–1378)
G3 postoperative complications	
Yes	22
No	80
cT factor (7th)	
1a/1b	5 / 31
2	25
3	30
4a/4b	4 / 7
cN factor (7th)	
0	45
1	36
2	19
3	2
cStage (7th)	
I (IA, IB)	27 / 11
II (IIA, IIB)	5 / 19
III (IIIA, IIIB, IIIC)	13 / 9 / 10
IV	8
p T factor (7th)	
0	6
1a/1b	9 / 33
2	20
3	27
4a/4b	5 / 2
p N factor (7th)	
0	49
1	28
2	15
3	10
p Stage (7th)	
0	3
I (IA, IB)	32 / 5
II (IIA, IIB)	3 / 16
III (IIIA, IIIB, IIIC)	19 / 8 / 11
IV	5
Location of tumor	
Cervical Esophagus	4
Upper thoracic esophagus	19
Middle thoracic esophagus	46
Lower thoracic esophagus	20
Abdominal esophagus	1
Esophagogastric junction	12
MAC scale	
Fighting spirit	47.8 (27–60)
Helpless/ Hopeless	9.2 (6–24)
Anxious Preoccupation	22.6 (13–32)
Fatalism	20.2 (8–30)
Avoidance	1.6 (1–4)
HADS, time 1	
≤ 10	65
≥ 11	37 (36.2%)
HADS, time 2	
≤ 10	61
≥ 11	41 (40.2%)
HADS, time 3	
≤ 10	54
≥ 11	48 (47.1%)
HADS, time 4	
≤ 10	58
≥ 11	44 (43.1%)
HADS, time 5	
≤ 10	67
≥ 11	35 (34.3%)

*BMI* body mass index, *c* clinical, *EORTC* European Organization for Research and Treatment, *HADS* Hospital Anxiety and Depression Scale, *HALS* hand-assisted laparoscopic surgery, *MAC* Mental Adjustment to Cancer, *p* pathological, *VATS* video-assisted thoracoscopic surgery

### Independent risk factors for psychological distress in bivariate analysis

We put variables to be provided with each 5 points into analysis. The 31 variables entered into univariate analysis were as follows: age; sex; history of cancer, surgery, alcohol consumption, or smoking; current smoking status (Brinkman index); operative procedure; operation time; intraoperative blood loss; postoperative complications (≥ grade 3); location of tumor; clinical (c-) T factor; c-N factor; c-stage; pathological (p-) T factor; p-N factor; p-stage; and MAC scale scores (fighting spirit, helpless/hopeless, anxious preoccupation, fatalism, and avoidance). All these variables are the perioperative information including the operative findings accumulating regularly at our hospital. The result of univariate analysis with each 5 points showed Additional files 4, 5, 6, 7, 8: Tables S1, S2, S3, S4, S5. The variables with a *p*-value less than 0.05 in univariate analysis were entered into bivariate logistic analysis.

The independent risk factors for psychological distress at times 1–5 is shown in Table 2. Independent predictors of psychological distress at time 1 were fighting spirit (OR 0.836, 95% CI 0.762–0.918; *p* < 0.001) and anxious preoccupation (OR 1.482, 95% CI 1.256–1.748; *p* < 0.001). Independent predictors at time 2 were fighting spirit (OR 0.885, 95% CI 0.803–0.977; *p* = 0.015) and helpless/ hopeless (OR 1.337, 95% CI 1.099–1.626; p = 0.004). At times 3–5, independent predictors were helpless/ hopeless. At time 4, final disease staging after esophagectomy (OR 2.379, 95% CI 1.430–3.957; *p* = 0.001) and fighting spirit (OR 0.913, 95% CI 0.847–0.983; *p* = 0.016) was identified as an independent risk factor.

**Table 2:** Risk factors for psychological distress at Time 1 to Time 5

Variables	Odds ratio	95% CIs	*p*-value
**Time 1**			
MAC; Fighting spirits	0.836	0.762–0.918	< 0.001
;Anxious preoccupation	1.482	1.256–1.748	< 0.001
**Time 2**			
MAC; Fighting spirits	0.911	0.837–0.991	0.029
; Helpless/ Hopeless	1.337	1.099–1.626	0.004
**Time 3**			
MAC; Helpless/ Hopeless	1.599	1.313–1.948	< 0.001
History of surgery	3.496	1.272–9.604	0.015
**Time 4**			
p-Stage (7th)	2.379	1.430–3.957	0.001
MAC; Fighting spirits	0.913	0.847–0.983	0.016
; Helpless/Hopeless	1.309	1.112–1.541	0.001
**Time 5**			
MAC; Helpless/Hopeless	1.575	1.303–1.905	< 0.001

*MAC* Mental Adjustment to Cancer, *CIs* Confidence interval

### Performance of these analyses

The C-index values for these bivariate analyses at times 1–5 are summarized in Table 3 and the five ROC curves are shown in Additional file 1: (see online Additional files). The area under the curve was 0.864 at time 1, 0.826 at time 2, 0.839 at time 3, 0.830 at time 4, and 0.840 at time 5. The internal consistency (reliability) of each scale was estimated by Cronbach's alpha coefficient. A value of 0.70 or greater was considered acceptable for group comparison [27]. In this study, the Cronbach's alpha coefficient between MAC scale and some variables of HADS scale in several point of perioperative treatment were Time 1: fighting spirits (α = 0.750), anxious preoccupation (α = 0.602); Time 2: fighting spirits (α = 0.702), helpless/ hopeless (α = 0.673); Time 3: helpless/ hopeless (α = 0.592); Time 4: fighting spirits (α = 0.838), helpless/ hopeless (α = 0.630); Time 5; helpless/ hopeless (α = 0.662).

**Table 3:** Evaluation of the predictive ability of the risk model at times 1–5 (N = 102)

Treatment time points	C index	95% CI	*p*-value
Time 1	0.864	0.792–0.936	< 0.001
Time 2	0.826	0.741–0.911	< 0.001
Time 3	0.839	0.757–0.920	< 0.001
Time 4	0.830	0.749–0.911	< 0.001
Time 5	0.840	0.759–0.920	< 0.001

*C index* concordance index, *CI* confidence interval

## Discussion

In this study, we evaluated the association between stress coping strategy and psychological distress with esophageal cancer from the time of the outpatient consultation (time 1) through to 3 months after esophagectomy (time 5). Our results showed that the relationship between psychological distress and psychological factors, such as Coping strategy, was stronger than that between psychological distress and individual patient characteristics. The C-index for these analyses showed high predictive performance at times 1–5. This is the first study to use prospective clinical data for the relation between the psychological distress and Coping strategy on each points of the treatment of esophageal cancer and may serve as a baseline for future research.

In the past report, Hellstadius et al. [28] reported that the proportion of patients with anxiety was 33% prior to surgery, 28% at 6 months, and 37% at 12 months and depression was 20% prior to surgery, 27% at 6 months, and 32% at 12-month. He concluded that anxiety symptoms remained stable over time whereas depression symptoms appeared to increase from pre-surgery to 6 months, levelling off between 6 and 12 months. On the other hand, this report showed that the proportion of patients with psychological distress was 36.2% in outpatients clinic, 40.2% prior to surgery, 47.1% at 14 days after surgery, 43.1% at 1 month, and 34.3% at 3 months (Table 1). In our study, the results were a little different that psychological distress appeared to increase from outpatient clinic to about 14 days after surgery, and gradually decreases from one month to 3 months after surgery. This study is the first report that examined the risk factors and psychological reactions or conditions in each point of treatment in more detail from outpatient clinic to 3 months after surgery.

In the past report, there were significant associations between the Coping strategies and psychological distress [13–15]. Therefore, there is a possibility that the Coping strategy improves the psychological distress. In another report, how patients cope with and adjust to threats is reportedly associated with depression [12]. However, it is still unclear whether the Coping strategies act on the psychological distress effectively in the various situations of the episode of the esophageal cancer treatment. Therefore, we investigated in more details the association between stress coping strategy and psychological distress on each points of the treatment of esophageal cancer.

Patients with a helpless/hopeless response and anxious preoccupation were at increased risk of psychological distress during the course of treatment for esophageal cancer, whereas those with a fighting spirit response were better able to adjust to their situations [13, 14]. Previous studies of the association between psychological distress and Coping strategy have suggested that the most beneficial response is fighting spirit and the most deleterious response may be helpless/ hopeless [11, 15, 25]. On the other hand, Petticrew et al. reported that the association between fighting spirit and psychological distress was not confirmed by larger study [29]. In esophageal cancer patients, maintaining a positive focus Coping strategies appears to minimize psychological harm in the past report [17, 30, 31]. However these reports didn’t describe such advantage on each points of the treatment of esophageal cancer. Therefore, it is yet unclear if such advantage can be observed on every point of the treatment or only on some limited phase. In the present study, helpless/ hopeless response was at increased risk of psychological distress at almost all the situations (times 2 to time 5). Anxious preoccupation was risk factor focusing on Coping strategy only at time 1. On the other hand, patients with a fighting spirit had fewer symptoms of anxiety and depression at the time of diagnosis in the outpatient clinic (time 1), at the determination of clinical stage (time 2), and at the determination of final staging (time 4). Recent advances in surgical techniques and perioperative intensive care have reduced the mortality and complications associated with esophagectomy, but it continues to be a challenging procedure with a reported mortality rate of 2.9–3.0% and a postoperative complication rate of 42.8–50.0% [2, 6, 8]. These esophageal cancer patients felt depressed and expressed fear of metastases and death during the course of treatment for esophageal cancer. In this study, we described that the deleterious Coping strategy such as helpless/ hopeless and anxious preoccupation had affected the psychological response all the situation of the treatments. On the other hand, the beneficial Coping strategy such as fighting spirit affected the psychological distress at the important situations for patients including of first outpatients clinic, determination of clinical stage, and determination of final stage after esophagectomy. On the other hand, although fighting spirit and anxious preoccupation were strongly related to psychological distress before treatment, as time of treatment passes, helpless/ hopeless was strongly related to psychological distress after esophagectomy. Therefore, we do not have to force patients to fighting spirit after esophagectomy, and also it is important to recognize and understand that feeling of helpless/hopeless may appear in these patients. In brief, we have to be responsible for continuous support so that patients do not give up a fight against esophageal cancer until the last. The two other adjustments styles such as fatalism and avoidance were not significant risk factors of psychological distress in this study.

We found that pathological staging was a significant risk factor of psychological distress at the determination of final staging after esophagectomy (time 4). It was reported that there is an association between psychological distress and tumor staging in the past report [32]. This is considered to be caused by the patient’s regret at allowing cancer to progress to an advanced stage, which interferes with the coping process. Previous research on emotional outcomes after resection for esophageal cancer found that a substantial proportion of patients who were alive at 1 year expressed fear of metastasis and death (80%) [17]. Therefore, we strongly recommend the development of a supportive care method for reducing anxiety and stress coping with esophageal cancer that as progressed to an advanced stage.

We expected before this study that the patients with history of surgery might be hard to feel depressed than the patients without history of surgery. However, we found that history of surgery was a significant risk factor of psychological distress but only at 1 month postoperatively before final staging (time 3). One possible explanation to this is that these patients may have felt postoperative agony from the gap with the former operation and the esophagectomy which is one of the most invasive in gastrointestinal surgery. Another explanation can be made that the esophagectomy in patients with previous abdominal or thoracic surgery tend to be more complex and time-consuming.

We already have a system in which various kinds of medical professionals such as gastroenterologists, surgeons, nurses, nutrients, dentists, otolaryngologists, and physical therapists, support esophageal cancer patients. However, psychiatrists and clinical psychologist have not been included in this team. For the patients having the predictive factors of the psychological distress, it would be very important to provide the mental intervention by psychiatrists before treatment. Therefore, we have to include psychiatrists and clinical psychologists in this esophageal team in our hosptal. And also, we hope that these medical teams are organized in other hospitals of Japan. More specific and personalized mental intervention will be needed in the near future.

Esophageal cancer is associated with substance dependence such as alcohol as reviewers comments. In this report, there were no relationships between psychological distress and drunker/ smoker. However, factors related to substance dependence influence coping style. In considering psychological support, it is important for medical professionals to prevent subsequent re-smoking and polydipsia in order to prevent recurrence, in addition to support related to cancer treatment.

This study has some limitations, in particular its single-center design, restricted nationality to Japanese, and small sample size. An external validation study or an international multicenter trial with a larger number of cases is needed to confirm our observations. There were also several factors potentially associated with psychological distress related to treatment for esophageal cancer that could not be controlled for this study. Nevertheless, our results confirm that the risk of psychological distress can be estimated reasonably accurately using the clinical factors investigated in this study. Our preliminary risk analysis could therefore be useful for risk stratification in actual clinical settings. Prospective accumulation of clinical data using this analysis could provide important information for better psychological management of patients undergoing treatment for esophageal cancer.

## Conclusion

This study showed that the relationship between psychological distress and Coping strategies was stronger on each points of the treatment of esophageal cancer than that between psychological distress and individual patient characteristics. This study uses basic clinical data and may provide the baseline information for risk stratification for psychological management and for future clinical studies in these patients.

## Supplementary Information

**Additional file 1. **Receiver operating characteristic curve for psychological distress in our risk model at times 1-5. (**a**) time 1, before definitive diagnosis. (**b**) time 2, after determination of clinical stage. (**c**) time 3, postoperatively before final staging. (**d**) time 4, determination of final stage at 1 month after esophagectomy. (**e**) time 5, 3 months after esophagectomy.

**Additional file 2. **Hospital Anxiety and Depression Scale (HADS).

**Additional file 3. **Mental Adjustment to Cancer (MAC) scale.

**Additional file 4: Table S1.** Risk factors for psychological distress at time 1.

**Additional file 5: Table S2.** Risk factors for psychological distress at time 2.

**Additional file 6: Table S3.** Risk factors for psychological distress at time 3.

**Additional file 7: Table S4.** Risk factors for psychological distress at time 4.

**Additional file 8: Table S5.** Risk factors for psychological distress at time 5.

## Data Availability

The datasets generated and analyzed during the current study are not publicly available given restrictions to data sharing imposed, but de-identified data are available from the corresponding author on reasonable request.

## Abbreviations

MAC scale: The mental adjustment to cancer scale
HADS: Hospital anxiety and depression scale
ORs: Odds ratios
95% CTs: 95% Confidence intervals
ROC curve: Receiver-operating characteristic curve
