# Supplementary material for: Association between psychological distress of each points of the treatment of esophageal cancer and stress coping strategy
Source: BMC Psychol. 2022 Sep 6;10:214. doi: 10.1186/s40359-022-00914-5 (PMC9450358; doi:10.1186/s40359-022-00914-5)

Supplemental Figure

Receiver-operating characteristic curve for psychological distress in our risk model at times 1-5.

(a) time 1, before definitive diagnosis

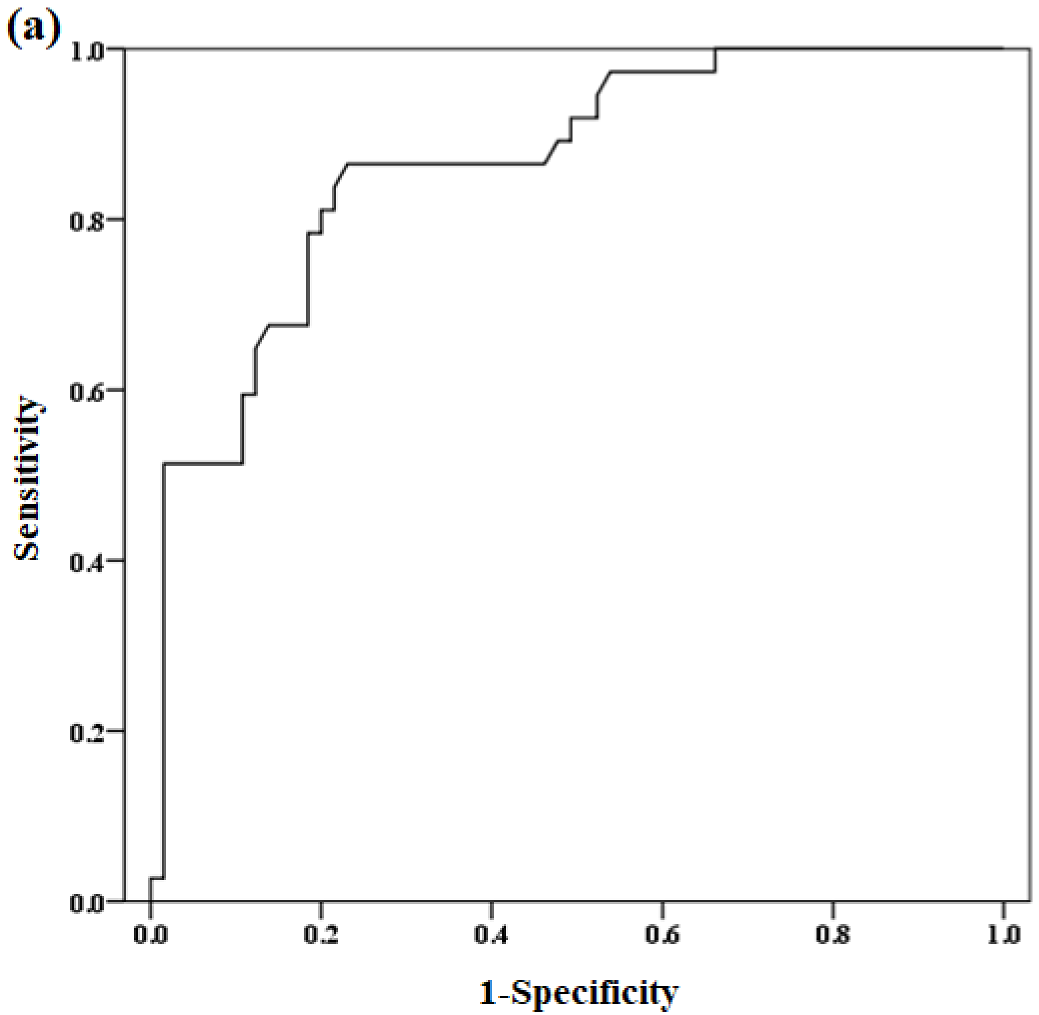

(b) time 2, after determination of clinical stage

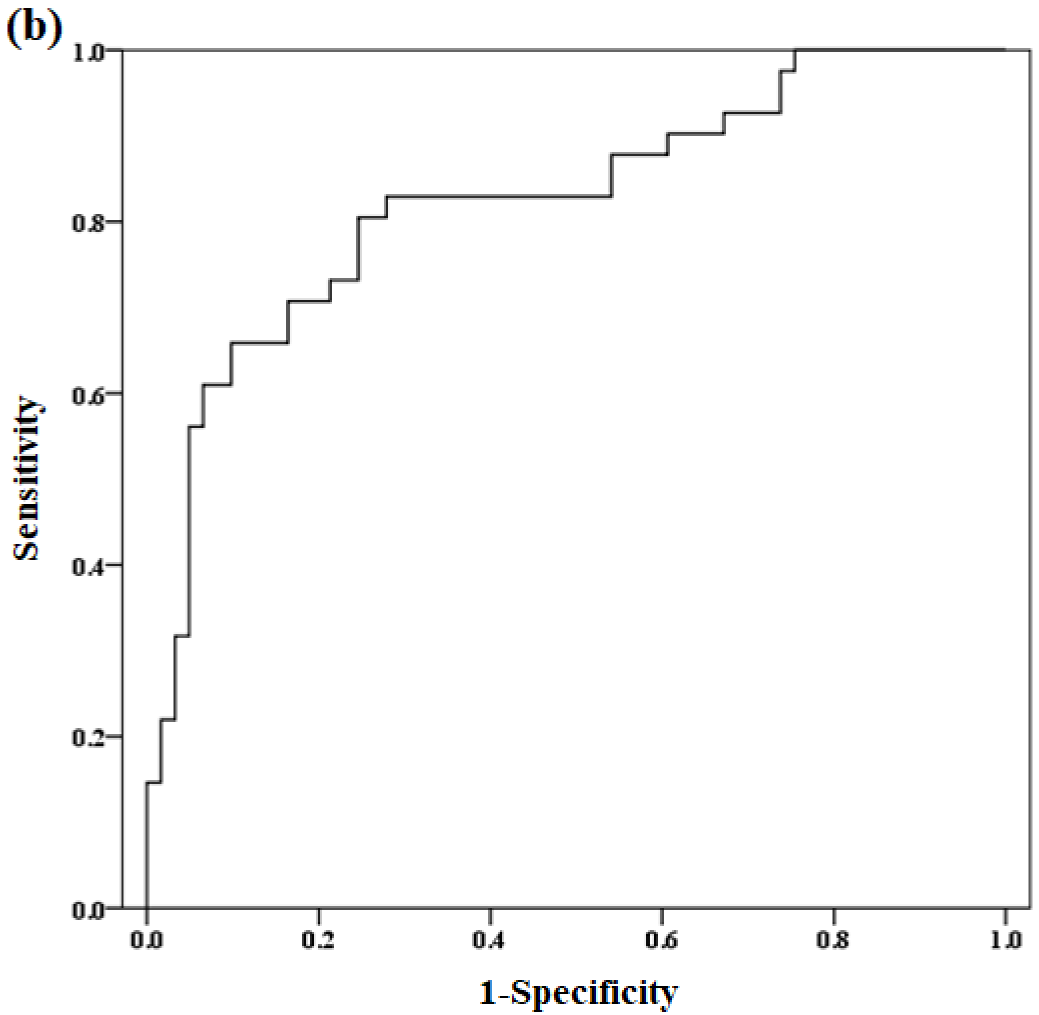

(c) time 3, postoperatively before final staging

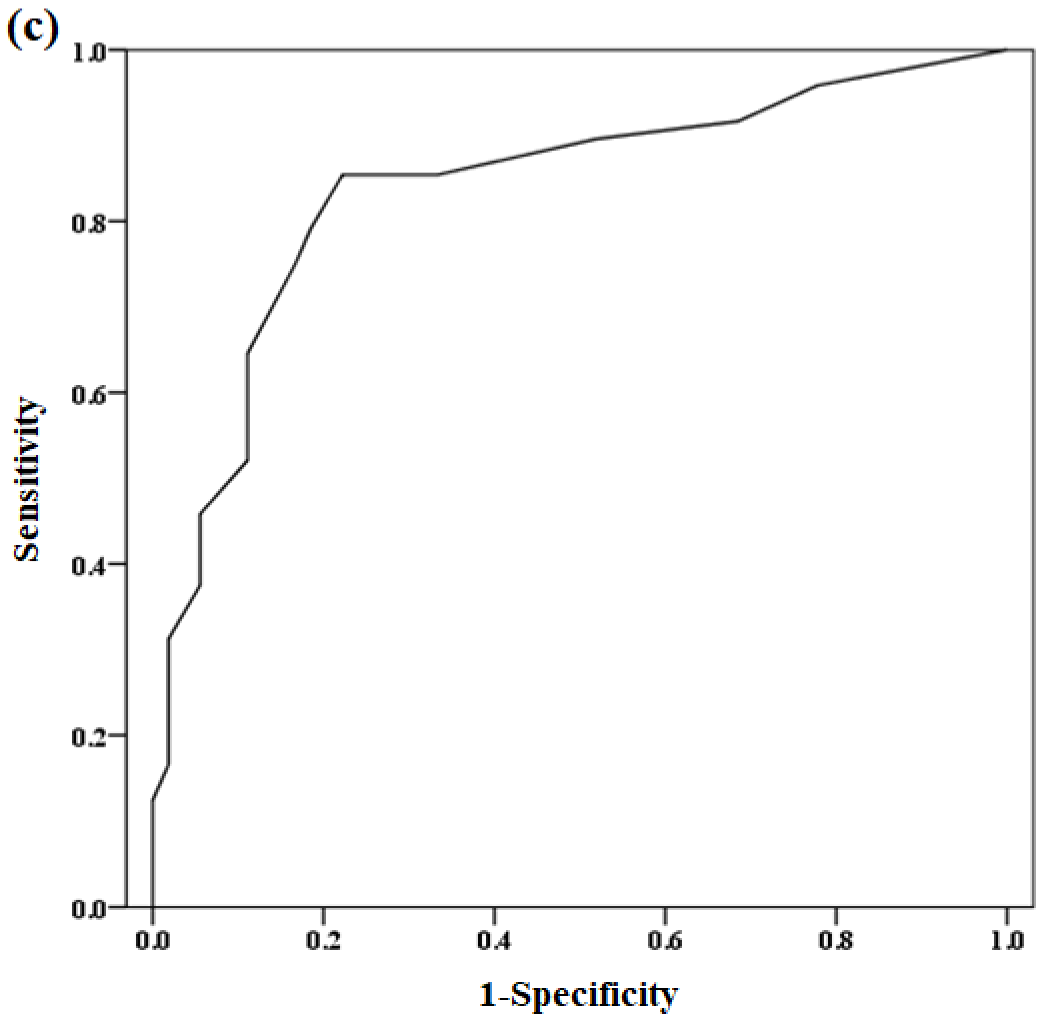

(d) time 4, determination of final stage at 1 month after esophagectomy

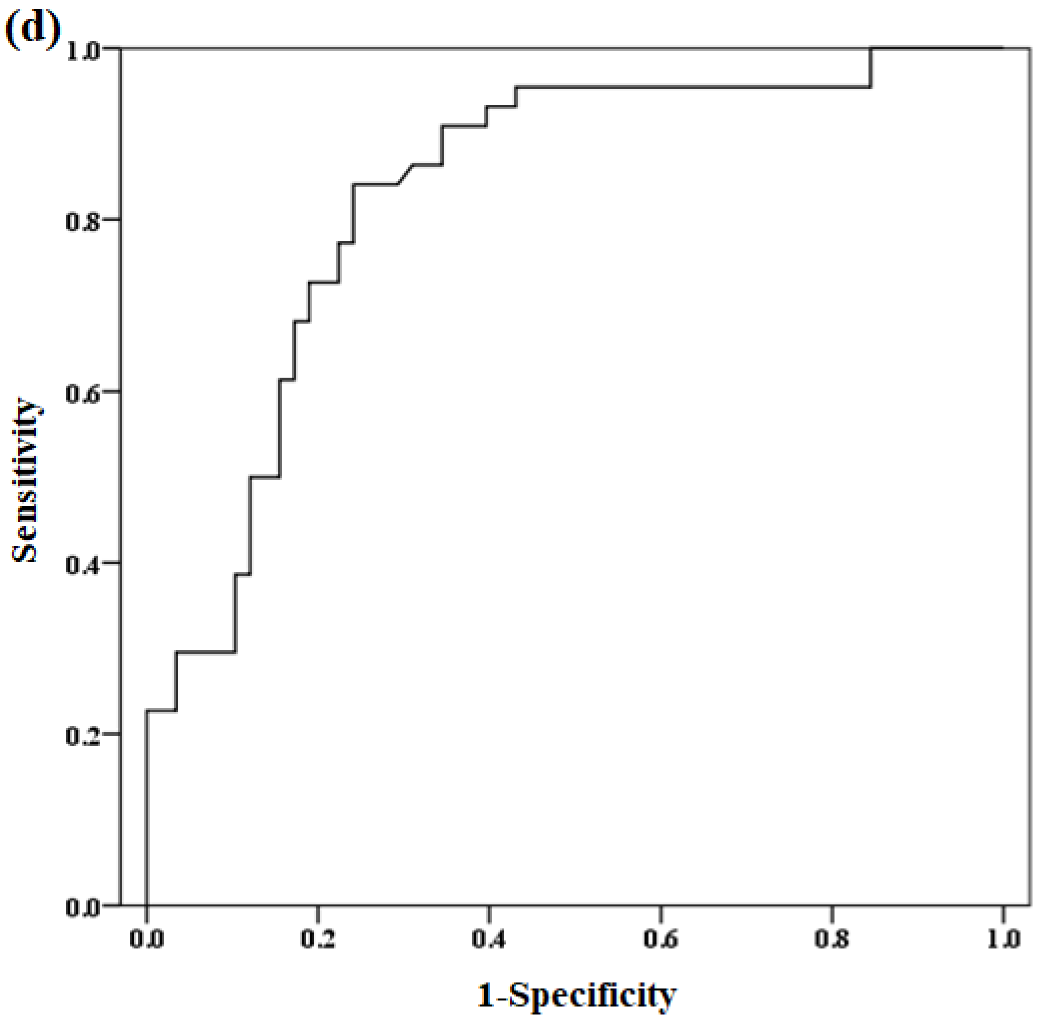

(e) time 5, 3 months after esophagectomy

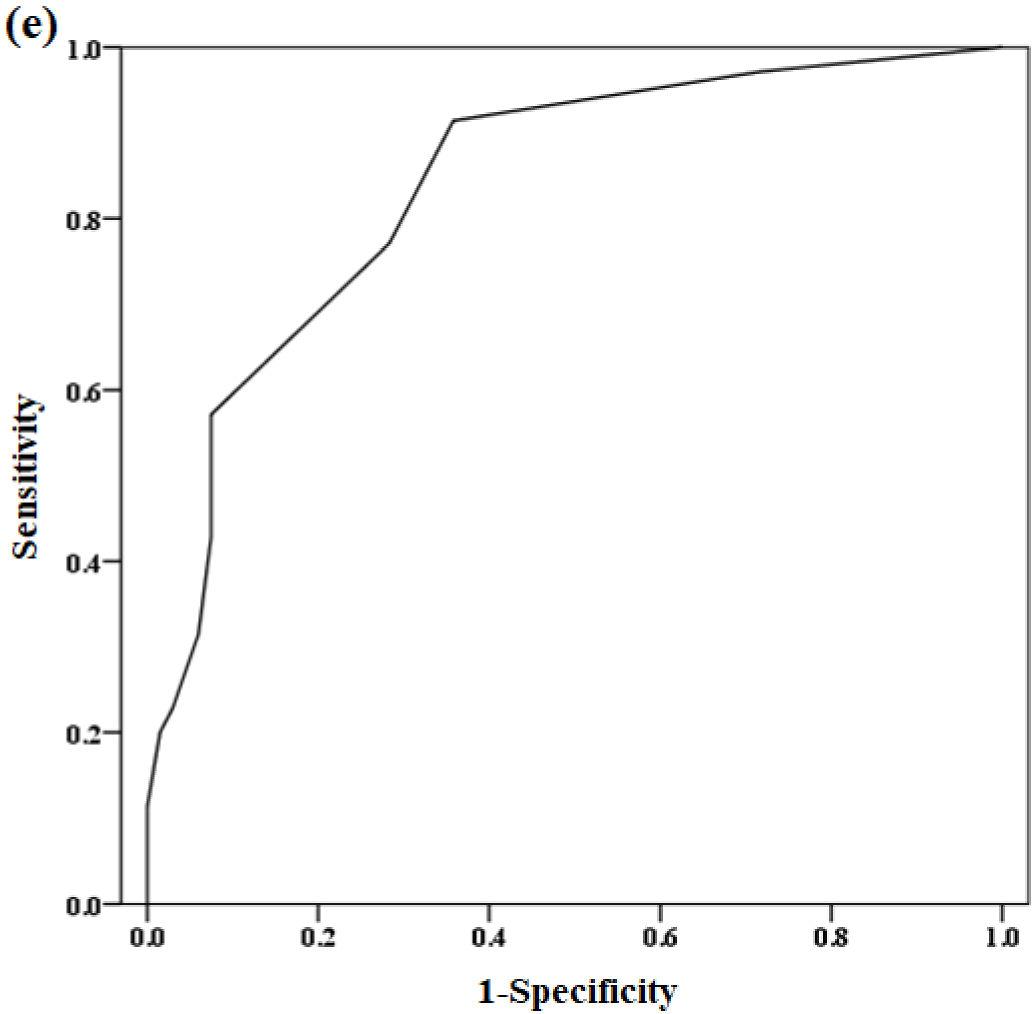

Supplement: Supplementary file 1 — Additional file 1. Receiver operating characteristic curve for psychological distress in our risk model at times 1-5. (a) time 1, before definitive diagnosis. (b) time 2, after determination of clinical stage. (c) time 3, postoperatively before final staging. (d) time 4, determination of final stage at 1 month after esophagectomy. (e) time 5, 3 months after esophagectomy. [file 40359_2022_914_MOESM1_ESM.pdf]
