# Supplementary material for: Association between psychological distress of each points of the treatment of esophageal cancer and stress coping strategy
Source: BMC Psychol. 2022 Sep 6;10:214. doi: 10.1186/s40359-022-00914-5 (PMC9450358; doi:10.1186/s40359-022-00914-5)
Supplement: Supplementary file 2 — Additional file 2. Hospital Anxiety and Depression Scale (HADS). [file 40359_2022_914_MOESM2_ESM.docx]

Hospital Anxiety and Depression Scale (HADS)

Instructions: Doctors are aware that emotions play an important part in most illnesses. If your doctor knows about these feelings, he or she will be able to help you more. This questionnaire is designed to help your doctor know how you feel. Read each item and place a firm tick in the box opposite the reply that comes closest to conveying how you have been feeling in the past week. Don’t take too long to think over your replies; your immediate reaction to each item will probably be more accurate than a long, thought-out response.

1. I feel tense or ‘wound up’:

　1　　Most of the time

　2　　A lot of the time

　3　　Time to time, occasionally

　4　　Not at all

1. I still enjoy the things I use to enjoy:

　1　　Definitely as much

　2　　Not quite so much

　3　　Only a little

　4　　Not at all

1. I get a sort of frightened feeling like something awful is about to happen:

　1　　Very definitely and quite badly

　2　　Yes, but not too badly

　3　　A little, but it doesn’t worry me

　4　　Not at all

1. I can laugh and see the funny side of things:

　1　　As much as I always could

　2　　Not quite so much now

　3　　Definitely not so much now

　4　　Not at all

1. Worrying thoughts go through my mind:

　1　　A great deal of the time

　2　　A lot of the time

　3　　From time to time but not too often

　4　　Only occasionally

1. I feel cheerful:

　1　　Not at all

　2　　Not often

　3　　Sometimes

　4　　Most of the time

1. I can sit at ease and feel relaxed:

　1　　Definitely

　2　　Usually

　3　　Not often

　4　　Not at all

1. I feel as if I have slowed down:

　1　　Nearly all of the time

　2　　Very often

　3　　Sometimes

　4　　Not at al

1. I get a sort of frightened feeling like having butterflies in my stomach:

　1　　Not at all

　2　　Occasionally

　3　　Quite often

　4　　Very often

1. I have lost interest in my appearance:

　1　　Definitely

　2　　I don’t take as much care as I should

　3　　I may not take quite as much care as I used to

　4　　I take just as much care as ever

1. I feel restless, as if I have to be on the move

　1　　Very much indeed

　2　　Quite a lot

　3　　Not very much

　4　　Not at all

1. I look forward with enjoyment to things:

1　　A much as I ever did

　2　　Rather less than I used to

　3　　Definitely less than I used to

　4　　Hardly at all

1. I get sudden feelings of panic:

　1　　Very often indeed

　2　　Quite often

　3　　Not very often

　4　　Not at all

1. I can enjoy a good book or radio or TV program:

　1　　Often

　2　　Sometimes

　3　　Not often

　4　　Very seldom
