# Supplementary material for: Association between psychological distress of each points of the treatment of esophageal cancer and stress coping strategy
Source: BMC Psychol. 2022 Sep 6;10:214. doi: 10.1186/s40359-022-00914-5 (PMC9450358; doi:10.1186/s40359-022-00914-5)
Supplement: Supplementary file 3 — Additional file 3. Mental Adjustment to Cancer (MAC) scale. [file 40359_2022_914_MOESM3_ESM.docx]

Mental Adjustment to Cancer (MAC) scale

Name: Date:

A number of statements are given below that describe people’s reactions to having cancer. Please mark the appropriate number to the right of each statement, indicating how much it applies to you at present. For example, if the statement definitely does not apply to you, then please mark 1 in the first column.

|  | Definitely does not apply to me | Does not apply to me | Applies to me | Definitely applies to me |
| --- | --- | --- | --- | --- |
| 1. I have been doing things that I believe will improve my health (e.g., I have changed my diet) | ［ 1 ］ | ［ 2 ］ | ［ 3 ］ | ［ 4 ］ |
| 1. I feel that I can’t do anything to cheer myself up | ［ 1 ］ | ［ 2 ］ | ［ 3 ］ | ［ 4 ］ |
| 1. I feel that problems with my health prevent me from planning ahead | ［ 1 ］ | ［ 2 ］ | ［ 3 ］ | ［ 4 ］ |
| 1. I believe that my positive attitude will benefit my health | ［ 1 ］ | ［ 2 ］ | ［ 3 ］ | ［ 4 ］ |
| 1. I don’t dwell on my illness | ［ 1 ］ | ［ 2 ］ | ［ 3 ］ | ［ 4 ］ |
| 1. I firmly believe that I will get better | ［ 1 ］ | ［ 2 ］ | ［ 3 ］ | ［ 4 ］ |
| 1. I feel that nothing I can do will make any difference | ［ 1 ］ | ［ 2 ］ | ［ 3 ］ | ［ 4 ］ |
| 1. I’ve left it all to my doctors | ［ 1 ］ | ［ 2 ］ | ［ 3 ］ | ［ 4 ］ |
| 1. I feel that life is hopeless | ［ 1 ］ | ［ 2 ］ | ［ 3 ］ | ［ 4 ］ |
| 1. I have been doing things that I believe will improve my health (e.g., exercise)[[This is the same as #1; please double check.]] | ［ 1 ］ | ［ 2 ］ | ［ 3 ］ | ［ 4 ］ |
| 1. Since my cancer diagnosis, I now realize how precious life is, and I’m making the most of it | ［ 1 ］ | ［ 2 ］ | ［ 3 ］ | ［ 4 ］ |
| 1. I’ve put myself in the hands of God | ［ 1 ］ | ［ 2 ］ | ［ 3 ］ | ［ 4 ］ |
| 1. I have plans for the future (e.g. holiday jobs, housing) | ［ 1 ］ | ［ 2 ］ | ［ 3 ］ | ［ 4 ］ |
| 1. I worry about the cancer returning or getting worse | ［ 1 ］ | ［ 2 ］ | ［ 3 ］ | ［ 4 ］ |
| 1. I’ve had a good life—what’s left is a bonus | ［ 1 ］ | ［ 2 ］ | ［ 3 ］ | ［ 4 ］ |
| 1. I think my state of mind can make a lot of difference to my health | ［ 1 ］ | ［ 2 ］ | ［ 3 ］ | ［ 4 ］ |
| 1. I feel that there is nothing I can do to help myself | ［ 1 ］ | ［ 2 ］ | ［ 3 ］ | ［ 4 ］ |
| 1. I try to carry on my life as I’ve always done | ［ 1 ］ | ［ 2 ］ | ［ 3 ］ | ［ 4 ］ |
| 1. I would like to make contact with others in the same boat | ［ 1 ］ | ［ 2 ］ | ［ 3 ］ | ［ 4 ］ |
| 1. I am determined to put it all behind me | ［ 1 ］ | ［ 2 ］ | ［ 3 ］ | ［ 4 ］ |
| 1. I have difficulty in believing that this has happened to me | ［ 1 ］ | ［ 2 ］ | ［ 3 ］ | ［ 4 ］ |
| 1. I suffer great anxiety about it | ［ 1 ］ | ［ 2 ］ | ［ 3 ］ | ［ 4 ］ |
| 1. I am not very hopeful about the future | ［ 1 ］ | ［ 2 ］ | ［ 3 ］ | ［ 4 ］ |
| 1. At the moment, I take one day at a time | ［ 1 ］ | ［ 2 ］ | ［ 3 ］ | ［ 4 ］ |
| 1. I feel like giving up | ［ 1 ］ | ［ 2 ］ | ［ 3 ］ | ［ 4 ］ |
| 1. I try to have a sense of humor about it | ［ 1 ］ | ［ 2 ］ | ［ 3 ］ | ［ 4 ］ |
| 1. Other people worry about me more than I do | ［ 1 ］ | ［ 2 ］ | ［ 3 ］ | ［ 4 ］ |
| 1. I think of other people who are worse off | ［ 1 ］ | ［ 2 ］ | ［ 3 ］ | ［ 4 ］ |
| 1. I am trying to get as much information as I can about cancer | ［ 1 ］ | ［ 2 ］ | ［ 3 ］ | ［ 4 ］ |
| 1. I feel that I can’t control what is happening | ［ 1 ］ | ［ 2 ］ | ［ 3 ］ | ［ 4 ］ |
| 1. I try to have a very positive attitude | ［ 1 ］ | ［ 2 ］ | ［ 3 ］ | ［ 4 ］ |
| 1. I keep quite busy, so I don’t have time to think about it | ［ 1 ］ | ［ 2 ］ | ［ 3 ］ | ［ 4 ］ |
| 1. I avoid learning more about it | ［ 1 ］ | ［ 2 ］ | ［ 3 ］ | ［ 4 ］ |
| 1. I see my illness as a challenge | ［ 1 ］ | ［ 2 ］ | ［ 3 ］ | ［ 4 ］ |
| 1. I feel fatalistic about it | ［ 1 ］ | ［ 2 ］ | ［ 3 ］ | ［ 4 ］ |
| 1. I feel completely at a loss about what to do | ［ 1 ］ | ［ 2 ］ | ［ 3 ］ | ［ 4 ］ |
| 1. I feel very angry about what has happened to me | ［ 1 ］ | ［ 2 ］ | ［ 3 ］ | ［ 4 ］ |
| 1. I don’t really believe that I have cancer | ［ 1 ］ | ［ 2 ］ | ［ 3 ］ | ［ 4 ］ |
| 1. I count my blessings | ［ 1 ］ | ［ 2 ］ | ［ 3 ］ | ［ 4 ］ |
| 1. I try to fight the illness | ［ 1 ］ | ［ 2 ］ | ［ 3 ］ | ［ 4 ］ |
