# Supplementary material for: Association between psychological distress of each points of the treatment of esophageal cancer and stress coping strategy
Source: BMC Psychol. 2022 Sep 6;10:214. doi: 10.1186/s40359-022-00914-5 (PMC9450358; doi:10.1186/s40359-022-00914-5)
Supplement: Supplementary file 4 — Additional file 4: Table S1. Risk factors for psychological distress at time 1. [file 40359_2022_914_MOESM4_ESM.docx]

Supplemental table 1. Risk factors for psychological distress at time 1

| Time 1 | HADS≤10  (n=65) | HADS≥11  (n=37) | *p-value* | *Hazard ratio* | *p-value* |
| --- | --- | --- | --- | --- | --- |
| Age: median (range) | 68.2(48–81) | 67.8(44–86) | 0.626 |  |  |
| Sex  　Male  Female | 56  9 | 30  7 | 0.498 |  |  |
| BMI | 22.7(15.0–32.1) | 20.7(14.1–41.9) | 0.016 |  |  |
| History of cancer  　 Yes  　 No | 16  49 | 8  29 | 0.732 |  |  |
| History of surgery  　 Yes  No | 21  44 | 15  22 | 0.403 |  |  |
| History of alcohol consumption  　 Yes  No | 58  7 | 30  7 | 0.410 |  |  |
| History of smoking  Yes  No | 57  8 | 30  7 | 0.365 |  |  |
| Brinkmann index | 610(0–2820) | 625(0–3040) | 0.875 |  |  |
| BI  <600  ≥600 | 27  38 | 17  20 | 0.666 |  |  |
| MAC scale (FS) | 50.0(29–60) | 44.4(27–55) | 0.005 | 0.836  (0.762–0.918) | <0.001 |
| MAC scale (H) | 7.9 (6–16) | 11.4 (6–24) | <0.001 |  |  |
| MAC scale (AP) | 21.0(13–31) | 24.8(17–32) | <0.001 | 1.482  (1.256–1.748) | <0.001 |
| MAC scale (F) | 19.1 (8–29) | 21.1(12–30) | 0.017 |  |  |
| MAC scale (A) | 1.5(1–4) | 1.6(1–4) | 0.578 |  |  |
