# Supplementary material for: Association between psychological distress of each points of the treatment of esophageal cancer and stress coping strategy
Source: BMC Psychol. 2022 Sep 6;10:214. doi: 10.1186/s40359-022-00914-5 (PMC9450358; doi:10.1186/s40359-022-00914-5)
Supplement: Supplementary file 5 — Additional file 5: Table S2. Risk factors for psychological distress at time 2. [file 40359_2022_914_MOESM5_ESM.docx]

Supplemental table 2. Risk factors for psychological distress at time 2

| Time 2 | HADS≤10  (n=61) | HADS≥11  (n=41) | *p-value* | *Hazard ratio* | *p-value* |
| --- | --- | --- | --- | --- | --- |
| Age: median (range) | 69.1 (48–81) | 67.0 (44–86) | 0.280 |  |  |
| Sex  　Male  Female | 54  7 | 32  9 | 0.154 |  |  |
| BMI | 23.0 (16.6–32.1) | 21.3 (14.1–41.9) | 0.001 |  |  |
| History of cancer  　 Yes  No | 15  46 | 9  32 | 0.758 |  |  |
| History of surgery  　 Yes  No | 19  42 | 17  24 | 0.285 |  |  |
| History of alcohol consumption  　 Yes  No | 55  6 | 33  8 | 0.088 |  |  |
| History of smoking  Yes  No | 52  9 | 35  6 | 0.987 |  |  |
| Brinkmann index | 622 (0–2820) | 560 (0–3040) | 0.790 |  |  |
| BI  <600  ≥600 | 23  38 | 21  20 | 0.177 |  |  |
| cT factor (7th)  1a  1b  2  3  4a  4b | 2  23  17  17  0  2 | 3  8  8  13  4  5 | 0.022 |  |  |
| cN factor (7th)  0  1  2  3 | 32  19  10  0 | 13  17  9  2 | 0.089 |  |  |
| cStage (7th)  I (IA, IB)  II (IIA, IIB)  III (IIIA, IIIB, IIIC)  IV | 18/10  3/11  8/6/1  4 | 9/1  2/8  5/3/9  4 | 0.027 |  |  |
| Tumor Localization  Ce  Ut  Mt  Lt  Ae  EGJ | 3  9  24  15  1  9 | 1  10  22  5  0  3 | 0.263 |  |  |
| Preoperative therapy  Yes  No | 37  24 | 28  13 | 0.432 |  |  |
| MAC scale (FS) | 50.0 (34–60) | 44.2 (27–60) | 0.006 | 0.911  (0.837–0.991) | 0.029 |
| MAC scale (H) | 7.8 (6–16) | 11.6 (6–24) | <0.001 |  |  |
| MAC scale (AP) | 21.3 (13–32) | 24.4 (14–32) | 0.001 | 1.337  (1.099–1.626) | 0.004 |
| MAC scale (F) | 18.8 (8–29) | 21.6 (12–30) | 0.004 |  |  |
| MAC scale (A) | 1.5 (1–4) | 1.8 (1–4) | 0.102 |  |  |
