# Supplementary material for: Association between psychological distress of each points of the treatment of esophageal cancer and stress coping strategy
Source: BMC Psychol. 2022 Sep 6;10:214. doi: 10.1186/s40359-022-00914-5 (PMC9450358; doi:10.1186/s40359-022-00914-5)
Supplement: Supplementary file 6 — Additional file 6: Table S3. Risk factors for psychological distress at time 3. [file 40359_2022_914_MOESM6_ESM.docx]

Supplemental table 3. Risk factors for psychological distress at time 3

| Time 3 | HADS≤10  (n=54) | HADS≥11  (n=48) | *p-value* | *Hazard ratio* | *p-value* |
| --- | --- | --- | --- | --- | --- |
| Age: median (range) | 68.0 (48–84) | 68.1 (44–86) | 0.665 |  |  |
| Sex  　Male  Female | 46  8 | 40  8 | 0.797 |  |  |
| BMI | 22.6 (17.4–27.7) | 21.7 (14.1–41.9) | 0.125 |  |  |
| History of cancer  　 Yes  No | 9  45 | 15  33 | 0.083 |  |  |
| History of surgery  　 Yes  No | 14  40 | 22  26 | 0.036 | 3.496  (1.272–9.604) | 0.015 |
| History of alcohol consumption  　 Yes  No | 48  6 | 40  8 | 0.176 |  |  |
| History of smoking  Yes  No | 44  10 | 43  5 | 0.249 |  |  |
| Brinkmann index | 660 (0–3040) | 530 (0–2820) | 0.607 |  |  |
| BI  <600  ≥600 | 20  34 | 24  24 | 0.187 |  |  |
| Thoracic approach  　VATS  　OPEN  　None | 49  3  2 | 39  6  3 | 0.370 |  |  |
| Abdominal approach  　HALS  　OPEN  　Lapa | 23  9  22 | 24  15  9 | 0.036 |  |  |
| Lymphadenectomy  　D0/1  　D2  　D3 | 3  20  31 | 2  12  34 | 0.388 |  |  |
| Curability  R0  R1/2 | 52  2 | 44  4 | 0.160 |  |  |
| Reconstruction  Gastric tube  Ileocolonic  Other | 36  13  5 | 30  13  5 | 0.908 |  |  |
| Thoracic duct  Resection  Preserve | 27  27 | 35  13 | 0.018 |  |  |
| Reconstruction route  Retrosternal  Posterior mediastinum | 45  9 | 43  5 | 0.360 |  |  |
| Operation time (min) | 590 (304–734) | 605 (213–774) | 0.499 |  |  |
| Bleeding time (ml) | 141 (25–1175) | 275 (25–1378) | 0.043 |  |  |
| Postoperative complication G3  Yes  No | 12  46 | 11  33 | 0.606 |  |  |
| cT factor (7th)  1a  1b  2  3  4a  4b | 4  23  18  10  1  2 | 1  8  7  20  3  5 | 0.003 |  |  |
| cN factor (7th)  0  1  2  3 | 34  16  8  0 | 11  20  11  2 | 0.005 |  |  |
| cStage (7th)  I (IA, IB)  II (IIA, IIB)  III (IIIA, IIIB, IIIC)  IV | 19/11  3/11  6/4/2  2 | 8/0  2/8  7/5/8  6 | 0.005 |  |  |
| Tumor Localization  Ce  Ut  Mt  Lt  Ae  EGJ | 3  9  25  9  1  11 | 1  10  21  11  0  1 | 0.102 |  |  |
| Preoperative therapy  Yes  No | 32  22 | 33  15 | 0.320 |  |  |
| MAC scale (FS) | 50.6 (34–60) | 44.7 (27–60) | 0.005 |  |  |
| MAC scale (H) | 7.4 (6–16) | 11.2 (6–24) | <0.001 | 1.599  (1.313–1.948) | <0.001 |
| MAC scale (AP) | 21.2 (13–32) | 24.1 (14–32) | 0.004 |  |  |
| MAC scale (F) | 18.7 (8–29) | 21.3 (12–30) | 0.007 |  |  |
| MAC scale (A) | 1.5 (1–4) | 1.7 (1–4) | 0.366 |  |  |
