# Supplementary material for: Association between psychological distress of each points of the treatment of esophageal cancer and stress coping strategy
Source: BMC Psychol. 2022 Sep 6;10:214. doi: 10.1186/s40359-022-00914-5 (PMC9450358; doi:10.1186/s40359-022-00914-5)
Supplement: Supplementary file 7 — Additional file 7: Table S4. Risk factors for psychological distress at time 4. [file 40359_2022_914_MOESM7_ESM.docx]

Supplemental table 4. Risk factors for psychological distress at time 4

| Time 4 | HADS≤10  (n=58) | HADS≥11  (n=44) | *p-value* | *Hazard ratio* | *p-value* |
| --- | --- | --- | --- | --- | --- |
| Age: median (range) | 68.3 (44–86) | 68.0 (53–79) | 0.984 |  |  |
| Sex  　Male  Female | 50  8 | 36  8 | 0.546 |  |  |
| BMI | 22.7 (16.6–27.7) | 21.4 (14.1–41.9) | 0.022 |  |  |
| History of cancer  　 Yes  No | 12  46 | 12  32 | 0.438 |  |  |
| History of surgery  　 Yes  No | 17  41 | 19  25 | 0.147 |  |  |
| History of alcohol consumption  　 Yes  No | 50  8 | 38  6 | 0.643 |  |  |
| History of smoking  Yes  No | 48  10 | 39  5 | 0.406 |  |  |
| Brinkmann index | 622 (0–3040) | 590 (0–2820) | 0.821 |  |  |
| BI  <600  ≥600 | 23  35 | 21  23 | 0.415 |  |  |
| Thoracic approach  　VATS  　OPEN  　None | 51  3  4 | 37  6  1 | 0.205 |  |  |
| Abdominal approach  　HALS  　OPEN  　Lapa | 23  12  23 | 24  12  8 | 0.065 |  |  |
| Lymphadenectomy  　D0/1  　D2  　D3 | 3  18  37 | 2  14  28 | 0.699 |  |  |
| Curability  R0  R1/2 | 57  1 | 39  4 | 0.087 |  |  |
| Reconstruction  Gastric tube  Ileocolonic  Other | 38  13  7 | 28  13  3 | 0.544 |  |  |
| Thoracic duct  Resection  Preserve | 25  33 | 37  7 | <0.001 |  |  |
| Reconstruction route  Retrosternal  Posterior mediastinum | 47  11 | 41  3 | 0.077 |  |  |
| Operation time (min) | 592 (213–774) | 589 (319–727) | 0.981 |  |  |
| Bleeding time (ml) | 178 (25–1175) | 258 (25–1378) | 0.285 |  |  |
| Postoperative complication G3  Yes  No | 9  21 | 10  28 | 0.737 |  |  |
| cT factor (7th)  1a  1b  2  3  4a  4b | 1  11  7  9  0  2 | 2  9  8  14  2  3 | 0.702 |  |  |
| cN factor (7th)  0  1  2  3 | 14  11  5  0 | 12  15  9  2 | 0.390 |  |  |
| cStage (7th)  I (IA, IB)  II (IIA, IIB)  III (IIIA, IIIB, IIIC)  IV | 11  6  11  2 | 10  10  14  4 | 0.002 |  |  |
| pStage (7th)  I (IA, IB)  II (IIA, IIB)  III (IIIA, IIIB, IIIC)  IV | 11  6  11  2 | 10  10  14  4 | 0.002 | 2.379  (1.430–3.957) | 0.001 |
| Tumor Localization  Ce  Ut  Mt  Lt  Ae  EGJ | 2  6  8  9  1  4 | 1  7  20  7  0  3 | 0.298 |  |  |
| Preoperative therapy  Yes  No | 31  27 | 34  10 | 0.013 |  |  |
|  |  |  |  |  |  |
| MAC scale (FS) | 50.1 (34–60) | 44.8 (27–57) | 0.007 | 0.913  (0.847–0.983) | 0.016 |
| MAC scale (H) | 7.8 (6–16) | 11.0 (6–24) | <0.001 | 1.309  (1.112–1.541) | 0.001 |
| MAC scale (AP) | 21.6 (13–31) | 23.9 (14–32) | 0.053 |  |  |
| MAC scale (F) | 19.3 (8–29) | 21.3 (12–30) | 0.050 |  |  |
| MAC scale (A) | 1.5 (1–4) | 1.7 (1–4) | 0.381 |  |  |
