# Supplementary material for: Association between psychological distress of each points of the treatment of esophageal cancer and stress coping strategy
Source: BMC Psychol. 2022 Sep 6;10:214. doi: 10.1186/s40359-022-00914-5 (PMC9450358; doi:10.1186/s40359-022-00914-5)
Supplement: Supplementary file 8 — Additional file 8: Table S5. Risk factors for psychological distress at time 5. [file 40359_2022_914_MOESM8_ESM.docx]

Supplemental table 5. Risk factors for psychological distress at time 5

| Time 5 | HADS≤10  (n=67) | HADS≥11  (n=35) | *p-value* | *Hazard ratio* | *p-value* |
| --- | --- | --- | --- | --- | --- |
| Age: median (range) | 68.0 (44–86) | 68.1 (53–79) | 0.714 |  |  |
| Sex  　Male  Female | 57  10 | 29  6 | 0.770 |  |  |
| BMI | 22.4 (15.2–27.7) | 21.5 (14.1–41.9) | 0.098 |  |  |
| History of cancer  　 Yes  No | 13  54 | 11  24 | 0.174 |  |  |
| History of surgery  　 Yes  No | 22  45 | 14  21 | 0.472 |  |  |
| History of alcohol consumption  　 Yes  No | 59  8 | 32  3 | 0.048 |  |  |
| History of smoking  Yes  No | 57  10 | 30  5 | 0.931 |  |  |
| Brinkmann index | 605 (0–3040) | 638 (0–2820) | 0.450 |  |  |
| BI  <600  ≥600 | 29  38 | 15  20 | 0.967 |  |  |
| Thoracic approach  　VATS  　OPEN  　None | 60  4  3 | 28  5  2 | 0.347 |  |  |
| Abdominal approach  　HALS  　OPEN  　Lapa | 28  14  25 | 19  10  6 | 0.109 |  |  |
| Lymphadenectomy  　D0/1  　D2  　D3 | 3  22  42 | 2  10  23 | 0.626 |  |  |
| Curability  R0  R1/2 | 66  1 | 30  5 | 0.023 |  |  |
| Reconstruction  Gastric tube  Ileocolonic  Other | 43  18  6 | 23  8  4 | 0.862 |  |  |
| Thoracic duct  Resection  Preserve | 33  34 | 29  6 | 0.001 |  |  |
| Reconstruction route  Retrosternal  Posterior mediastinum | 57  10 | 31  4 | 0.626 |  |  |
| Preoperative treatment  Yes  No | 40  27 | 25  10 | 0.242 |  |  |
| Operation time (min) | 592 (213–774) | 597 (319–727) | 0.893 |  |  |
| Bleeding time (ml) | 170 (25–1175) | 286 (25–1378) | 0.042 |  |  |
| Postoperative complication G3  Yes  No | 14  53 | 9  26 | 0.737 |  |  |
| cT factor (7th)  1a  1b  2  3  4a  4b | 4  25  17  16  2  3 | 1  6  8  14  2  4 | 0.580 |  |  |
| cN factor (7th)  0  1  2  3 | 36  20  11  0 | 9  16  8  2 | 0.018 |  |  |
| cStage (7th)  I (IA, IB)  II (IIA, IIB)  III (IIIA, IIIB, IIIC)  IV | 21/11  3/10  10/5/3  4 | 6/0  2/9  3/4/7  4 | 0.020 |  |  |
| Tumor Localization  Ce  Ut  Mt  Lt  Ae  EGJ | 3  10  29  14  1  10 | 1  9  17  6  0  2 | 0.538 |  |  |
| Preoperative therapy  Yes  No | 11  4 | 18  12 | 0.378 |  |  |
| MAC scale (FS) | 50.1 (34–60) | 44.5 (27–57) | 0.007 |  |  |
| MAC scale (H) | 7.7 (6–16) | 12.0 (6–24) | <0.001 | 1.575  (1.303–1.905) | <0.001 |
| MAC scale (AP) | 21.8 (13–32) | 24.2 (14–32) | 0.054 |  |  |
| MAC scale (F) | 18.9 (8–29) | 22.1 (12–30) | 0.001 |  |  |
| MAC scale (A) | 1.5 (1–4) | 1.7 (1–4) | 0.399 |  |  |
